# Appropriateness of frequent use of emergency departments: A retrospective analysis in Rome, Italy

**DOI:** 10.3389/fpubh.2023.1150511

**Published:** 2023-04-04

**Authors:** Giuseppe Furia, Antonio Vinci, Vittoria Colamesta, Paolo Papini, Adriano Grossi, Vittoria Cammalleri, Patrizia Chierchini, Massimo Maurici, Gianfranco Damiani, Corrado De Vito

**Affiliations:** ^1^Department of Public Health and Infectious Diseases, Sapienza University of Rome, Rome, Italy; ^2^Local Health Authority Roma 1, Borgo Santo Spirito, Rome, Italy; ^3^Nursing Sciences and Public Health, University of Rome “Tor Vergata”, Rome, Italy; ^4^Department of Biomedicine and Prevention, University of Rome “Tor Vergata”, Rome, Italy; ^5^Section of Hygiene, University Department of Life Sciences and Public Health, Università Cattolica del Sacro Cuore, Rome, Italy

**Keywords:** frequent user, emergency department, appropriateness, Local Health Authority, Italy, Rome, COVID-19

## Abstract

**Background:**

Frequent users (FUs) are patients who repeatedly and inappropriately visit the emergency department (ED) for low-grade symptoms that could be treated outside the hospital setting. This study aimed to investigate the phenomenon of the FU in Rome by profiling such users and analyzing ED attendance by FUs.

**Methods:**

The analysis was carried out for attendance in 2021 at 15 EDs in the Local Health Authority Roma 1 geographical area. A digital app collected data, including information on the following variables: number of attendance, demographic characteristics, emergency medical service (EMS) usage, triage code, and appropriateness of attendance. COVID-19 diagnosis was also studied to analyze any possible influence on ED attendance. Differences between FUs and non-FUs were investigated statistically by *t*-test and chi-square test. Univariate analysis and multivariable logistic regression were performed to analyze the associated factors.

**Results:**

A total of 122,762 ED attendance and 89,036 users were registered. The FU category represented 2.9% of all users, comprising 11.9% of total ED attendance. There was a three times higher frequency of non-urgent codes in attendance of FU patients (FU: 9.7%; non-FU: 3.2%). FUs were slightly more likely to have used the EMS (13.6% vs. 11.4%) and had a lower frequency of appropriate ED attendance (23.8% vs. 27.0%). Multivariate logistic analysis confirmed a significant effect of triage code, gender, age, EMS usage, and COVID-19 diagnosis for the appropriateness of attendance. The results were statistically significant (*p* < 0.001).

**Conclusion:**

The FU profile describes mostly non-urgent and inappropriate attendance at the ED, including during the COVID-19 pandemic. This study represents an important tool for strengthening preventive policies outside the hospital setting. The Italian National Recovery and Resilience Plan represents an excellent opportunity for the development of new strategies to mitigate the phenomenon of FUs.

## Background

In recent decades, the progressive aging of the population and the higher levels of urbanization and pollution are increasing the number of patients suffering from chronic diseases, which has a significant impact on the use of healthcare services, in particular emergency departments (EDs) (1, 2). In Italy, despite significant improvements in the emergency care system, the reduction in the number of hospital beds over the last 30 years, and the increase in the number of fragile patients have led to an increase in ED attendance (3, 4). Published evidence from many countries shows that frequent ED visit increases the risk of adverse effects such as hospitalization, functional decline, and complications related to treatment and procedures (1, 5, 6). A significant proportion of hospital attendance are inappropriate, and the respective ailments could be managed by non-acute healthcare services outside the hospital setting; such inappropriate attendance, therefore, drive up costs and increase inefficiency (7–9). The causes of frequent ED visit are multifactorial: Although many patients have chronic medical problems, these are often combined with marked psychosocial morbidity (10, 11). Social factors are also present in a large percentage of frequent users (FUs), including loneliness, poverty, poor quality of life, and difficulties in daily self-management (12–14).

Against this backdrop, the COVID-19 pandemic caused a significant decrease in overall ED visit, with reduced volumes of up to 50% in some countries (15). The largest proportional reduction in ED users was in preventable ED attendance, including accident and traumatic injuries, probably as a result of reduced motor vehicle travel and fewer work activities, but also to time-dependent illnesses, such as stroke or cardiac complaints, among older people, possibly due to concerns about COVID-19 acquisition in hospital (16, 17). In Italy and in other countries, the COVID-19 pandemic also influenced the use of emergency medical services (EMSs) (18–20).

Despite copious international literature on the frequent use of EDs, there is no single definition of a FU. The selection of threshold values is often subjective and is generally based on previous literature or the distribution of ED attendance in a given period. More often, a FU is considered a user with ≥4 or 5 attendance at the hospital for both physical and mental issues, but various alternatives, ranging from 2 to 12 attendance per year or 6-month period, have also been chosen (1, 3, 12, 21–24).

Other studies highlight the complexity of the needs of older adults, which increase the risk of readmission after discharge (25–27). Similar studies have also been conducted in Italy and the Netherlands, showing that while FUs represent a small percentage of hospital attendance, they nevertheless form a high proportion of the total costs of EDs (28–30).

In Italy, the availability of beds in the hospital for acute patients and outside the hospital for post-acute assistance is a major issue for the National Health System, especially in metropolitan areas such as Rome, the most populous municipality in Italy, where three large Local Health Authorities administer healthcare services. Various Italian studies have investigated the problem by analyzing some of the characteristics of the FU (2, 12, 24, 28) but, despite the importance of the issue, there is still insufficient consideration in the literature of the following points: number of attendance, usage of EMS, level of urgency, and appropriateness of attendance. The COVID-19 diagnosis was also investigated for a possible influence on ED attendance.

This study investigates ED attendance in Rome; it describes the characteristics of the FU population and defines a FU profile that highlights the differences between FUs and non-FUs and identifies factors linked to FU status and appropriate ED attendance.

## Materials and methods

### Study design and data collection

A retrospective cohort study was carried out during 2022 of the ED attendance in 2021, i.e., from 1 January 2021 to 31 December 2021. The Local Health Authority (LHA) Roma 1 geographical area in Rome was chosen for the analysis because it is one of the most populous areas in Italy, with 13 EDs (of 22 in the Rome metropolitan area) and ~878,000 residents aged >15 in 2021, with an aging index (number of population aged >64 years per 100 individuals aged < 14 years) of 192 (the Italian mean is 183.3) (31).

The study population included all patients with residency in the LHA Roma 1 geographical area who were admitted to any of its 13 local EDs. Attendance records from two other EDs close to the LHA Roma 1 area were added to the total to include potential ED attendance of LHA Roma 1 residents outside the main metropolitan area. A digital platform was used to extract the ED data from the Lazio region's official data flows for emergency attendance. The data are pseudo-anonymized: Although the ID code of each patient is represented by an encrypted string, it is still possible to connect health events attributable to the same individual. Using this pseudo-anonymized ID, subsequent attendance of the same individual in 2021 were counted and classified according to the number of attendance made.

Records included information on the following variables for each patient:

- Number of attendance: A FU is defined as having ≥4 attendance per year, according to the literature (10, 23–25, 29, 32, 33);
- Demographic characteristics: age and gender;
- Arrival mode: by EMS or not by EMS;
- Triage code: In 2001, a 24-h nurse-led triage system was introduced by the Italian Ministry of Health to evaluate a patient's level of urgency, with assessment resulting in the assignment of a priority code. Since 2019, a transition from color codes to numerical codes (1–5) has gradually been introduced (34);
- Appropriateness of attendance: According to a visiting physician evaluation, all patients, who were admitted to a hospital ward, had refused admission to a hospital ward, or died in the ED, were considered as appropriate;
- Diagnosis of COVID-19: defined by any positive swab during ED attendance.

Because all variables are mandatory in each patient's attendance record, there were no missing data.

Individuals < 16 years old and single-specialism EDs (ophthalmology, pediatrics, and obstetrics) were excluded from the study because they could affect the appropriateness of the results.

### Statistical analysis

Microsoft^®^ Excel^®^ v.2016 MSO and STATA v. 17.0 were used for data analysis. The cumulative number of ED attendance was computed for each patient ID, and patients with ≥4 attendance were classified as FUs.

Descriptive analysis was performed on all variables recorded. Descriptive statistics, such as mean, SD, frequency, and percentage, were used to describe the demographics and ED attendance characteristics of the sample.

For inferential analysis, given the large sample size, statistical significance was determined at a level of *p* = 0.001. Welch's *t*-test was used to compare mean ages between FUs and non-FUs. Pearson's chi-square test was used to investigate differences in categorical variables between FUs and non-FUs. Univariate analysis was performed for any potentially associated factor. Multivariable logistic regression was performed for all factors identified with the significance level set at *p* < 0.001.

## Results

During the study period, 89,036 patients residing in LHA Roma 1 had at least one attendance at one of the EDs investigated, for a total of 122,762 ED attendance (Table 1). In particular, 68,711 patients (77.2%) attended an ED only once, while 2,545 patients (2.9%) were considered FUs (≥4 attendance) and were responsible for 14,644 ED attendance (11.9% of total attendance). Among these FUs, 1,297 patients (1.5%) produced 5,188 ED attendance (4.2% of total attendance), and 38 patients had more than 20 ED attendance each, for a total of 1,406 ED attendance.

**Table 1:** Distribution of ED attendance and patients during 2021.

**Cumulative attendance**	**No. of patients**	**% of total patients**	**No. of attendance**	**% of total attendance**
1	68,711	77.17	68,711	55.97
2	13,933	15.65	27,866	22.70
3	3,847	4.32	11,541	9.40
4	1,297	1.46	5,188	4.23
5	535	0.60	2,675	2.18
6	252	0.28	1,512	1.23
7	155	0.17	1,085	0.88
8	71	0.08	568	0.46
9	60	0.07	540	0.44
10	46	0.05	460	0.37
11	17	0.02	187	0.15
12	23	0.03	276	0.22
13	18	0.02	234	0.19
14	14	0.02	196	0.16
15	5	0.01	75	0.06
16	4	0.00	64	0.05
17	4	0.00	68	0.06
18	4	0.00	72	0.06
19	2	0.00	38	0.03
≥20	38	0.04	1,406	1.15
Total patients:	89,036	100.00	122,762	100.00

ED, emergency department.

Demographic characteristics are summarized in Table 2. No gender difference was observed in the non-FU population, while there were more female subjects in the FU population (*p* < 0.001). Conversely, among non-FUs, female subjects attending an ED were significantly older than male subjects (mean age 56.2 vs. 52.3 for male subjects; *p* < 0.001), whereas no significant age difference was found among FU patients (mean age 56.9 vs. 56.5 for male subjects).

**Table 2:** Characteristics of ED users and attendance, stratifying by user demographic variables and ED attendance parameters.

**Variable**	**Population**			***P*-value**
	**Total patients (*****N*** = **89,036)**	**Non-FU patients (*****N*** = **86,491)**	**FU patients (*****N*** = **2,545)**	
**Gender**				
Male	44,050 (49.5%)	43,815 (50.7%)	1,171 (46.0%)	**< 0.001** ^*^
Female	44,986 (50.5%)	42,676 (49.3%)	1,374 (53.9%)	
**Age [mean (SD)]**				
Male	52.4 (SD 19.9)	52.3 (SD 19.9)	56.5 (SD 19.5)	0.30^**§**^ (FU)
Female	56.2 (SD 20.6)	56.2 (SD 20.6)	56.9 (SD 20.4)	**< 0.001**^**§**^ (non-FU)
	**Total attendance (*****N*** = **122,762)**	**Non-FU attendance (*****N*** = **108,118)**	**FU attendance (*****N*** = **14,644)**	
**Triage code**				
1	4,835 (3.9%)	3,417 (3.2%)	1,418 (9.7%)	**< 0.001** ^*^
2	46,272 (37.7%)	41,383 (38.3%)	4,889 (33.4%)	
3	42,989 (35.0%)	38,103 (35.2%)	4,886 (33.4%)	
4	22,456 (18.2%)	19,813 (18.3%)	2,643 (18.0%)	
5	6,210 (5.1%)	5,402 (5.0%)	808 (5.5%)	
**Arrival mode**				
EMS	14,644 (11.9%)	10,822 (11.4%)	3,822 (13.6%)	< 0.001*
NO EMS	108,118 (88.1%)	83,839 (88.6%)	24,279 (86.4%)	
**Appropriateness**				
Appropriate	32,674 (11.9%)	29,191 (27.0%)	3,483 (23.8%)	**< 0.001** ^*^
Not appropriate	90,088 (88.1%)	78,927 (73.0%)	11,161 (76.2%)	
**COVID-19 diagnosis**				
Yes	1,295 (1.1%)	1,216 (1.1%)	79 (0.5%)	**< 0.001** ^*^
No	121,467 (98.9%)	106,902 (98.9%)	14,565 (99.5%)	

^*^Pearson's chi-square test; ^**§**^Welch's t-test for the mean difference in gender (male vs. female), within non-FU and FU patients.

ED, emergency department; FU, frequent user; EMS, emergency medical service. Bold values indicate statistically significant *p*-value < 0.05.

Regarding the ED attendance triage code, 1,418 patients were assigned code 1, 4,889 patients code 2, 4,886 code 3, 2,643 code 4, and 808 code 5. Thus, there was a three times higher frequency of non-urgent codes for attendance of FU patients, compared to non-FUs (9.7% vs. 3.2%). FUs were slightly more likely to have used EMSs (13.6% vs. 11.4%), but they also had a lower frequency of appropriate ED attendance compared to non-FUs (23.8% vs. 27.0%). There were 79 (0.5%) COVID-19 diagnoses among FU attendance and 1,216 (1.1%) for non-FUs.

In Table 3, the logistic regression analysis results are described. In the univariable analysis, triage code, arrival mode, gender, and age were significantly associated with the appropriateness of attendance [OR = 2.94 (2.89–2.99), OR = 3.69 (3.59–3.80), OR = 1.05 (1.03–1.08), 1.03 (1.03–1.03), respectively]. On the contrary, the number of attendance in a year significantly reduced appropriateness [OR = 0.97 (0.97–0.98)]. The multivariate logistic analysis confirmed a significant effect of attendance triage code, gender, age, and EMS usage on the appropriateness of attendance, while the annual number of attendance was negatively associated with appropriateness. COVID-19 diagnosis was associated with more appropriate ED attendance.

**Table 3:** Logistic regression analysis between the appropriateness of ED attendance and attendance-associated factors.

**Variable**	**OR (95% CI)**	***P*-value**
**Univariable analysis**		
Number of attendance	0.97 (0.97–0.98)	< 0.001
Triage code (global)	2.94 (2.89–2.99)	< 0.001
2	1.45 (1.28–1.64)	
3	6.65 (5.89–7.50)	
4	15.57 (13.79–17.58)	
5	37.99 (33.34–43.29)	
Arrival mode (by EMS)	3.69 (3.59–3.80)	< 0.001
Gender (male)	1.05 (1.03–1.08)	< 0.001
Age (years)	1.03 (1.03–1.03)	< 0.001
COVID-19 diagnosis	0.02 (0.02–0.03)	< 0.001
**Multivariable analysis** ^*^		
Number of attendance	0.97 (0.97–0.98)	< 0.001
Triage code (global)	2.45 (2.39–2.47)	< 0.001
2	1.37 (1.21–1.56)	
3	5.23 (4.62–5.92)	
4	11.40 (10.07–12.92)	
5	31.16 (27.26–35.62)	
Arrival mode (by EMS)	1.14 (1.12–1.18)	< 0.001
Gender (male)	1.03 (1.02–1.03)	< 0.001
Age (years)	1.81 (1.75–1.87)	< 0.001
COVID-19 diagnosis	11.23 (9.78–12.90)	< 0.001

^*^Constant: 0.01 (0.01–0.01). p < 0.001.

ED, emergency department; EMS, emergency medical service.

## Discussion

### Main results

The descriptive analysis shows the distribution of patients per number of ED attendance during 2021. In line with the international literature, a very small number of patients are responsible for a large share of the attendance, which contribute to the overcrowding of EDs (24, 35–38). A comparison of the proportion of FUs in different studies is complicated by the different FU definitions used, particularly with respect to the number of ED attendance. The prevalence of FUs thus ranges from 1 to 20% of total ED users, but they can account for more than 60% of the total visit volume (22, 39–44). In this study, FUs are defined as patients recording ≥4 attendance and represent 2.8% of total patients but accounted for 11.9% of total attendance; these results are similar to those of another Italian study (24). Considering the wide variability of results due to limitations and different methodologies used, these percentages are higher (23, 32, 33) or lower (22, 45, 46) in the other studies. The demographic characteristics describe the FU as most likely male subjects, aged < 65, in agreement with other studies (10, 23, 32, 33). Most FUs accessed the ED by EMS but were not assigned an urgent triage code or diagnosed with an urgent condition that required hospitalization, confirming international trends, which also pertained during the COVID-19 pandemic (2, 10, 12, 25, 47–49). There has been scarce evidence about the impact of the COVID-19 pandemic on the use of ED services among frequent ED users. In this study, COVID-19 diagnosis would seem not to be associated with frequent usage of ED services but was associated with the appropriateness of attendance. While this needs more analysis and further investigation, it is possible that the less-severe cases were well managed at the primary care level, through rapid alerts and notification of cases, and through collaboration between public health services and general practitioners.

The inappropriate frequent use of EDs revealed in this study, as demonstrated by the relatively large number of attendance per individual FU, may have a marked impact on healthcare capacity and resource management (14); indeed, it could lead to overcrowding of facilities and, consequently, compromise the quality of ED services, while also increasing the risk of human error (1, 29, 30, 50).

Despite the difficulty of comparing different healthcare systems across the world, there is a broad consensus that some targeted interventions on FUs can reduce hospital attendance and therefore can also lead to economic and organizational savings. Individual and personalized programs of care for FUs can reduce the organizational and economic impact on hospitals, as well as improve the clinical and social outcomes of patients.

An OECD proposal to meet population health needs outside the hospital setting has already been accepted in a number of countries (51), and some studies have shown how the strengthening of primary care services (e.g., extending primary care opening hours or timely palliative care) (52, 53) may mitigate the FU phenomenon and help to reduce the use of EDs (2, 54, 55), especially among older adults and frail members of the population, who may have to cope with different levels of functional limitation (56). Previous systematic reviews of interventions for frequent ED users have described patient-centered programs predicated on interdisciplinary team approaches. Interventions such as continuous case management and implementation of various care plans and social work visits with frequent follow-up are effective in reducing the cost of care and subsequent ED and inpatient visits (21, 57, 58). Health literacy and information campaigns may also help to improve health status and, consequently, reduce hospital and emergency attendance (59). Patients, in particular older adults, may have multiple barriers that prevent them from accessing primary care services. A critical cause of the increase in the number of non-urgent attendance may be long waiting lists largely due to the lack of a well-established primary care system (50). Furthermore, where health services require an appointment or a co-payment, the choice of a hospital ED that does not have these characteristics is likely to seem more attractive (60–62). Another point is the lack of primary care facilities, such as community hospitals, nursing homes, or rehabilitation clinics, for post-acute care; such facilities are especially important in the absence of caregivers and allow hospital wards to discharge more patients, guaranteeing appropriate transitional settings to patients moving from hospital to home and resulting in greater availability of inpatient beds, without compromising waiting lists for elective hospitalizations (7, 63, 64).

This report seems to be the first study to profile FUs in Italy during the COVID-19 pandemic and to analyze attendance at multiple EDs in a single metropolitan area. The population attending the EDs studied represents a large sample of the Roman ED population and thus forms an important starting point for understanding the FU phenomenon in Italy. This is the first study to analyze simultaneously the use of EMSs, level of urgency, COVID-19 diagnosis, and appropriateness of attendance in FUs. The ease of access to regional data flows and the use of a digital platform allowed rapid and efficient collection of official data, such that no record or information was missed.

This study has some limitations. This was a retrospective study, and for this reason, it was not possible to trace deaths during the study period or interventions by primary care services. While considering the attendance of two other EDs close to the LHA Roma 1 area, patients could have re-attended another ED outside the included network without being counted. The clinical and pharmacological characteristics of patients, as well as self-perceived variables, such as physical pain or mental distress that are associated with frequent use of EDs, were not studied here. Information about income and level of education is not provided in the regional databases. COVID-19 cases were identified by positive swab, but other variables would be necessary to quantify COVID-19-specific attendance together with their appropriateness, in particular where the COVID-19 diagnosis was made after admission to a hospital ward.

In this study, all patients who were admitted to the hospital wards or were waiting for a bed were considered to be appropriate attendance, although it is unknown whether these patients received the correct diagnosis. The appropriateness of admission may need a better definition in subsequent studies.

## Conclusion

Frequent use of the ED is a challenging and contentious issue for clinicians and policymakers. In Italy, this study is an important tool for anticipating the needs of specific categories of patients who might use alternative healthcare services rather than hospitals. In this context, one aim of the Italian National Recovery and Resilience Plan is to strengthen health districts and primary healthcare services using interventions, such as telemedicine, which played an important role during the COVID-19 pandemic and also to focus on the social support required by some categories of patients such as FUs. The Plan, therefore, represents an excellent opportunity to develop some of the strategies described in the scientific literature to mitigate the phenomenon of the FU. Before this, future studies should focus on the clinical and social heterogeneity of FUs, categorizing them into subgroups according to urban or rural origin, as well as the conditions and diseases that necessitate hospitalization.

## Data availability statement

The raw data supporting the conclusions of this article will be made available by the authors, without undue reservation.

## Ethics statement

Ethical review and approval was not required for the study on human participants in accordance with the local legislation and institutional requirements. Written informed consent for participation was not required for this study in accordance with the national legislation and the institutional requirements.

## Author contributions

GF contributed to literature search, study design, data analysis, result interpretation, and the write-up of the manuscript. AV, VCo, and AG were responsible for study design, statistical analysys, and result interpretation. PP and VCa was responsible for study design and results interpretation. PC, GD, and CD oversaw the project as the co-principal investigators and contributed to study design and result interpretation. All authors critically appraised and approved the manuscript.
